# All‐Solid‐State Garnet‐Based Lithium Batteries at Work–In Operando TEM Investigations of Delithiation/Lithiation Process and Capacity Degradation Mechanism

**DOI:** 10.1002/advs.202205012

**Published:** 2022-12-18

**Authors:** An‐Yuan Hou, Chih‐Yang Huang, Chih‐Long Tsai, Chun‐Wei Huang, Roland Schierholz, Hung‐Yang Lo, Hermann Tempel, Hans Kungl, Rüdiger‐A. Eichel, Jeng‐Kuei Chang, Wen‐Wei Wu

**Affiliations:** ^1^ Department of Materials Science and Engineering National Yang Ming Chiao Tung University Hsinchu 30010 Taiwan; ^2^ Institut für Energie– und Klimaforschung (IEK‐9: Grundlagen der Elektrochemie) Forschungszentrum Jülich D‐52425 Jülich Germany; ^3^ Department of Materials Science and Engineering Feng Chia University No. 100, Wenhwa Rd Seatwen Taichung 40724 Taiwan; ^4^ Institut für Materialien und Prozesse für elektrochemische Energiespeicher– und wandler RWTH Aachen University D‐52074 Aachen Germany; ^5^ Institut für Energie– und Klimaforschung (IEK–12: Helmholtz–Institute Münster Ionics in Energy Storage) Forschungszentrum Jülich D‐48149 Münster Germany; ^6^ Center for the Intelligent Semiconductor Nano‐system Technology Research Hsinchu 30078 Taiwan

**Keywords:** all‐solid‐state Li battery, garnet, in operando transmission electron microscopy, interface, Li_7_La_3_Zr_2_O_12_

## Abstract

Li_7_La_3_Zr_2_O_12_ (LLZO)‐based all‐solid‐state Li batteries (SSLBs) are very attractive next‐generation energy storage devices owing to their potential for achieving enhanced safety and improved energy density. However, the rigid nature of the ceramics challenges the SSLB fabrication and the afterward interfacial stability during electrochemical cycling. Here, a promising LLZO‐based SSLB with a high areal capacity and stable cycle performance over 100 cycles is demonstrated. In operando transmission electron microscopy (TEM) is used for successfully demonstrating and investigating the delithiation/lithiation process and understanding the capacity degradation mechanism of the SSLB on an atomic scale. Other than the interfacial delamination between LLZO and LiCoO_2_ (LCO) owing to the stress evolvement during electrochemical cycling, oxygen deficiency of LCO not only causes microcrack formation in LCO but also partially decomposes LCO into metallic Co and is suggested to contribute to the capacity degradation based on the atomic‐scale insights. When discharging the SSLB to a voltage of ≈1.2 versus Li/Li^+^, severe capacity fading from the irreversible decomposition of LCO into metallic Co and Li_2_O is observed under in operando TEM. These observations reveal the capacity degradation mechanisms of the LLZO‐based SSLB, which provides important information for future LLZO‐based SSLB developments.

## Graphical abstract

Li_7_La_3_Zr_2_O_12_‐based all‐solid‐state Li battery is made to deliver a high areal capacity and stable cycle performance. In operando transmission electron microscopy is performed to observe the charge/discharge processes while three capacity degradation mechanisms, that is, interface delamination, microcrack formation, and LiCoO_2_ decomposition, are revealed.

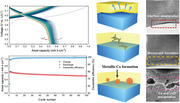

## 1 Introduction

Batteries have been attracting considerable attention in the field of energy storage owing to the increasing demand for renewable energy storage, mobile, and portable electronic applications.^[^ ^1^ ^]^ Among these systems, Li‐ion batteries (LIBs) are promising energy storage options because of their high energy densities, long cycle lives, and high operational voltages.^[^ ^2^, ^3^, ^4^, ^5^, ^6^, ^7^ ^]^ However, the use of flammable organic liquid electrolytes poses safety concerns,^[^ ^8^, ^9^ ^]^ while their poor thermal stability and high reactivity toward metallic Li decrease the potential of using metallic Li as the anode for achieving higher energy densities and electrochemical performances.^[^ ^10^, ^11^, ^12^ ^]^ Therefore, enhancing the safety and increasing the energy density of LIBs remain vital issues. In the past years, several methods, such as using smart batteries to predict the formation of Li dendrites and environmental cooling to dissipate excessive heat to protect batteries from thermal and mechanical abuse, have been developed to promote the safety and cycle life of LIBs.^[^ ^13^, ^14^ ^]^ Furthermore, the replacement of the organic liquid electrolyte with a solid one for making all‐solid‐state Li batteries (SSLBs) is a promising technique owing to the intrinsic safety of solid‐state electrolytes (SSEs) while the use of metallic Li as the anode can promote their energy densities.^[^ ^15^, ^16^ ^]^ Several common SSEs are used in SSLB research including garnet‐type Li_7_La_3_Zr_2_O_12_ (LLZO),^[^ ^17^, ^18^, ^19^ ^]^ sodium superionic conductor (NASICON)‐type Li_1.3_Al_0.3_Ti_1.7_(PO_4_)_3_ (LATP),^[^ ^20^, ^21^, ^22^ ^]^ phosphosulfide‐type Li_10_GeP_2_S_12_ (LGPS),^[^ ^23^, ^24^ ^]^ Li_3_PS_4_,^[^ ^25^, ^26^ ^]^ halide‐type Li_3_MCl*_x_* (M = In, Sc, Y, Zr) (*x* = 4, 6),^[^ ^27^, ^28^, ^29^, ^30^ ^]^ and argyrodite‐type Li_6_PS_5_X (X = Cl, Br, I).^[^ ^27^, ^31^ ^]^ Among these SSEs, phosphosulfide‐type,^[^ ^32^, ^33^, ^34^ ^]^ halide‐type,^[^ ^35^ ^]^ and argyrodite‐type^[^ ^36^ ^]^ solid electrolytes have relatively low elastic modulus than that for garnet‐type^[^ ^37^ ^]^ and NASICON‐type.^[^ ^38^, ^39^ ^]^ The advantage of SSEs with low elastic modulus is enabling SSLB fabrication by using cold‐pressing to establish Li‐ion and electronic conductive paths between the used materials, as well as the use of carbon‐based material to increase their electronic conductivities on the electrodes. The cold‐pressing process avoids material decomposition reactions or elemental interdiffusion, which can be triggered at high sintering temperatures, to achieve a low interface resistance and allow electrochemical cycling at high current densities. However, the drawback of using these types of materials is that it usually needs very high external pressure, from a couple of MPa up to hundreds of MPa, for achieving stable electrochemical cycling of the batteries.^[^ ^27^, ^29^, ^40^, ^41^, ^42^ ^]^ The necessity of high external pressure may retard the practical use of the battery or lower the overall energy density at the packing level of the battery. On the other hand, the higher elastic properties of LLZO^[^ ^37^ ^]^ and LATP^[^ ^38^ ^]^ usually need high sintering temperatures to establish proper Li‐ion and electronic conductive paths within the battery cell.^[^ ^18^, ^21^ ^]^ A good combination of materials and interfacial engineering for avoiding material decomposition reactions or elemental interdiffusion between the solid electrolyte and the active cathode (AC) material at the high sintering temperature becomes a challenge.^[^ ^43^, ^44^, ^45^, ^46^ ^]^ The disadvantage of high‐temperature sintering then dramatically impedes the development of SSLBs using LLZO and LATP as their solid electrolytes. Nevertheless, the high elastic property of LLZO and LATP may allow these types of SSLBs to cycle without applying external pressure, which offers the possibility of achieving higher energy density and safety than using lower elastic modulus ones.^[^ ^21^, ^47^ ^]^

LLZO has many unique properties than other SSEs such as a relatively high elastic modulus, wide electrochemical window, and high Li‐ion conductivity (≈1 mS cm^−1^ at 25 °C).^[^ ^48^ ^]^ Depending on the substitutions, LLZO also provides excellent electrochemical stability toward metallic Li that offers the potential for achieving higher energy density.^[^ ^49^, ^50^ ^]^ Therefore, many research groups have evinced a strong interest in developing LLZO‐based SSLBs. However, the development of LLZO‐based SSLB has been slow in progress. Challenges are majorly coming from the high interfacial resistances between the rigid materials, which impede the Li‐ion diffusion within the cell. On the negative electrode side, novel strategies, such as applying interlayer coatings and diminishing surface impurities, are proposed to achieve good contact between Li and LLZO to reduce the interfacial resistance to as low as several Ω cm^2^.^[^ ^48^, ^51^, ^52^, ^53^, ^54^, ^55^, ^56^ ^]^ The penetration of Li dendrite through LLZO solid electrolyte is then relieved but not yet completely solved. Therefore, extensive efforts are still put into fully understanding the fundamental mechanism of Li deposition/dissolution through LLZO solid electrolytes with the hope of completely suppressing the Li dendrite formation.^[^ ^57^, ^58^, ^59^, ^60^, ^61^, ^62^, ^63^, ^64^, ^65^ ^]^

On the positive electrode side, the rigid nature of both LLZO and AC materials requires a high‐temperature sintering process to establish Li‐ion and electronic conductive paths, thereby reducing the interfacial resistance and promoting the electrochemical performance of the battery.^[^ ^18^, ^21^, ^47^, ^66^, ^67^ ^]^ Since the Li‐ion conductivity of AC is usually very low, composited positive electrode (CPE) is proposed to build up a 3D percolation network for ionic and electronic transportation, where LLZO provides Li‐ion conductive paths and AC offers electronic and partially Li‐ion pathways. The 3D structure of CPE also increases the contact area between LLZO and AC for faster ionic diffusion pathways that allow the battery for achieving higher power density. From a material processing point of view, three different strategies are proposed to fabricate CPE that enables the successful cycle of the LLZO‐based SSLBs. Sintering additives, such as lithium‐borate (LBO)^[^ ^66^, ^68^, ^69^, ^70^ ^]^ and lithium‐borate‐carbonate (LBCO),^[^ ^47^ ^]^ are proposed to fabricate CPE at ≈700 °C by taking advantage of the low melting point of these sintering additives. The liquid phase of the sintering additive at ≈700 °C could effectively densify the CPE by capillary force while the relatively lower processing temperature avoids the formation of a highly resistive interface from the decomposition of materials. When cooling down to room temperature, the sintering additive serves as a cement to glue the LLZO and AC together, as well as offering Li‐ion conductive paths, due to its nature of low Li‐ion conductivity.

The second strategy is by going through material selections, for which LLZO and AC need to be chemically stable at/near their sintering temperatures.^[^ ^18^, ^19^ ^]^ For example, LiCoO_2_ (LCO) exhibits high chemical stability to LLZO up to 1085 °C and has a similar thermal expansion coefficient as that for LLZO, and offers relatively high electronic conductivity than other ACs. The combination of LLZO and LCO for CPE fabrication allows fast heating and cooling during the sintering process to 1050 °C without delamination from LLZO SSE. The sintering process triggers the neck growth of the ceramic particles to establish the 3D percolation paths for Li‐ion and electronic conductions, as well as to improve the contact and access of the electrochemically active surface area of AC. Therefore, a higher areal capacity and operational current density of the SSLB can be achieved by this method. Infiltration of AC into porous scaffold SSE to form CPE is the third suggested strategy.^[^ ^67^, ^71^ ^]^ The porous scaffold LLZO can be made by mixing LLZO with organic pore former to sinter at high temperatures. The organic pore former burns out at high temperatures to leave the well‐sintered LLZO as the backbone for providing a well‐connected 3D porous structure for Li‐ion conduction. Then, the AC precursor can be infiltrated into the LLZO porous scaffold to crystallize at a relatively lower temperature than the LLZO sintering temperature to form a well‐bonded interface. The advantage of this strategy is that the contact interface could be maximized by the infiltration of liquid phase AC precursor while the leftover porous after the crystallization of AC could tolerate the volume change of AC during electrochemical cycling.

Although the interface between LLZO and AC can be built up by the above‐mentioned three strategies, the capacity fading during electrochemical cycling of LLZO‐based SSLB is still considered high, especially when the areal loading of CPE is relatively high. An explanation for the capacity degradation is usually due to the loss of contact between LLZO and AC during the electrochemical cycling.^[^ ^18^, ^66^, ^72^ ^]^ By assuming the interface is electrochemically stable, the volume change of AC during the lithiation/delithiation process leads to the formation of tensile stress on the interface of SSE/AC, which eventually could result in interfacial delamination. It further leads to higher tortuosity for Li‐ion and electronic transportation, which raises the total resistance of the battery. Therefore, the charge/discharge voltage will hit the cut‐off voltage at a lower capacity for a fixed charge/discharge current density due to the higher cell resistance. This also explains why SSLBs that report lower areal AC loadings usually have higher capacity retention than the higher ones.

Hence, the LLZO‐based SSLB could be one of the most important technologies for energy storage applications in the future. An understanding of the capacity degradation mechanism is essential for further improvement of its electrochemical cycling performance. Especially, the failure mechanism on the interface of LLZO/AC during the delithiation/lithiation process was mostly investigated by the post‐mortem analysis method, where the delamination of AC from LLZO only can be concluded from the observation of separated materials.^[^ ^18^, ^42^, ^66^ ^]^ In this study, an LLZO‐based SSLB was fabricated by sintering LCO/LLZO CPE directly onto a dense LLZO pellet. By carefully optimizing the microstructure, the fabricated battery shows excellent electrochemical cycling performance over 100 cycles when metallic Li was used as the anode. Subsequently, in operando transmission electron microscopy (TEM) was conducted to demonstrate the successful delithiation/lithiation of the SSLB under TEM and investigate the well‐sintered interface between LCO and LLZO during electrochemical cycling. Interfacial delamination and material decomposition were observed during electrochemical cycling at different working voltages by using high‐resolution transmission electron microscopy (HRTEM). The capacity degradation mechanisms during cycling the LLZO‐based SSLB, which are essential for the further optimization of SSLB, have been discussed using this thorough analysis.

## 2 Results and Discussion

### 2.1 Electrochemical performance and characterization of SSLB

**Figure 1:**
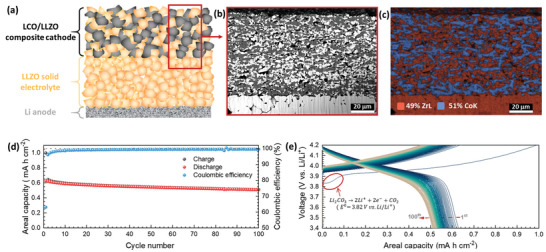
Microstructure analysis and electrochemical performance of the LLZO‐based SSLB. a) Sketch of LLZO‐based SSLB. b) SEM image of the CPE microstructure. c) EDS elemental mapping image of the CPE. The Co represents the LCO phase and Zr represents the LLZO phase. d) Electrochemical cycling performance of the LLZO‐based SSLB with a current density of 50 µA cm^−2^ at 60 °C. e) Charge/discharge curves of the LLZO‐based SSLB which are shown in (d).

The electrochemical performance and schematic illustration of the LLZO‐based SSLB are shown in **Figure** **1**. Figure 1a shows the sketch of fabricated bulk‐type LLZO‐based SSLB, where the LCO/LLZO mixture is used as the CPE, LLZO is used as the SSE, and metallic Li is used as the anode. After sintering, a typical CPE microstructure shows a well‐connected network of LLZO and LCO to provide conductive paths for Li‐ions and electrons, Figure 1b. When compared to the previous report by Tsai et al.,^[^ ^18^ ^]^ the microstructure of CPE in this battery is much more homogeneous with much fewer big pores. Energy dispersive spectrometer (EDS) elemental mapping shows clearly the separation of LCO and LLZO phases, which indicate that there was no severe chemical reaction during the sintering process (Figure 1c). Furthermore, the quantity analysis by EDS is in agreement with the prepared LLZO:LCO weight ratio to be 1:1. The electrochemical cycling performance between 4.2 and 3.4 V versus Li/Li^+^ with a charge/discharge current density of 50 µA cm^−2^ for the LLZO‐based SSLB is shown in Figure 1d. The first charge areal capacity was 1 mA h cm^−2^ (167 mA h g^−1^) while that for the first discharge was only 0.63 mA h cm^−2^ (105 mA h g^−1^), which gives the Coulombic efficiency for the first electrochemical cycle only 63%. Nevertheless, the Coulombic efficiency runs up to 96.1% in the 2nd cycle and stays around 99.5% for most of the rest cycles. After 100 cycles, the discharge capacity retention of the SSLB was 80.8%, which is much higher than 30.4% for the previous report.^[^ ^18^ ^]^ The SSLB exhibits one of the most stable cycling performances of LLZO‐based SSLB with a relatively high CPE loading. Figure 1e shows the charge/discharge curves of the cycled SSLB. The most noticeable is that the 1st charge curve had a much lower polarization and larger areal capacity than that of all the others. The capacity fading from 1st (167 mA h g^−1^) to 2nd (108 mA h g^−1^) charge cycle was 35.4%. When considering the theoretical capacity of Li*_x_* CoO_2_ from *x* = 1 to *x* = 0.5 is 147 mA h g^−1^, that is, charge to 4.2 V versus Li/Li^+^, the very high charge capacity indicates that there was some electrochemical decomposition of materials during the 1st charging process. Nevertheless, there was almost no measurable capacity at the voltage below 3.77 V versus Li/Li^+^ in the 1st charging curve, which makes the calculated oxidation potential of LLZO at 2.91 V versus Li/Li^+^ arguable.^[^ ^73^, ^74^ ^]^ The first plateau that appears on the 1st charging curve is between 3.77 and 3.92 V versus Li/Li^+^. In general, this plateau is not observed when using phosphosulfide‐type,^[^ ^24^, ^41^, ^42^ ^]^ halide‐type,^[^ ^27^, ^28^, ^29^ ^]^ and argyrodite‐type^[^ ^75^ ^]^ SSEs to fabricate SSLBs and was no more seen in 2nd charging cycle. Hence, it could be attributed to the electrochemical decomposition of Li_2_CO_3_, Li*_x_* O*_y_*, or LiOH, which were formed from Li‐proton exchange with the moisture in the air during the cooling process of the SSLB fabrication process.^[^ ^76^, ^77^, ^78^ ^]^ When subtracting the capacity from the decomposition of impurities, the total capacity of 1st charge gets close to the theoretical capacity of LCO. However, the capacity gained above 3.92 V versus Li/Li^+^ is still much higher than 2nd charging cycle. The loss of the capacity and the increase of polarization could be due to the delamination at LCO/LLZO interface and LCO decomposition as will be discussed in the later in operando TEM section. The discharge curves, which show a sharp increase of discharging slope at the voltage below 3.8 V versus Li/Li^+^, are similar to those batteries using liquid electrolyte and LCO cathode.^[^ ^79^, ^80^, ^81^ ^]^ The similarity in discharging curves indicates that the LCO within the CPE was well maintained in its phase and structure during the sintering process. Initial structural characterization is essential for the investigation of the dynamic delithiation and lithiation effects on SSLB. Figure S1a, Supporting Information, shows a schematic of the SSLB prepared by a focused ion beam (FIB) for in operando TEM investigation. X‐ray diffraction (XRD) was used for confirming the crystal structures of the bulk CPE before processing it into a TEM sample, which exhibits only the characteristic peaks of LCO and LLZO (Figure S1b, Supporting Information). In addition, Figure S1c, Supporting Information, shows the low‐magnification TEM image of the SSLB sample prepared by FIB, where Pt wires were deposited on the surface of the SSLB to connect the LCO and LLZO particles. In this case, Pt also served as the anode since it forms an alloy with metallic Li with oxidation peaks at 0.34 and 0.42 V versus Li/Li^+^.^[^ ^82^, ^83^, ^84^ ^]^ The brighter and darker contrasts of the image in Figure S1c, Supporting Information, indicate LCO and LLZO, respectively. Here, the crystal structure of cubic LLZO was also confirmed by HRTEM, which gives the d‐spacing 0.418 nm for LLZO_(103)_, (Figure S1d, Supporting Information). The corresponding electron diffraction pattern with a zone axis of [010] is shown in Figure S1e, Supporting Information.^[^ ^85^ ^]^

### 2.2 In operando TEM investigation of structural variation and failure phenomenon of SSLB

After a series of structural identification, the in operando delithiation/lithiation of the SSLB under TEM was conducted. Additionally, in the in operando observation, the working conditions of the in operando specimens should be as consistent as possible with the actual operation of SSLB. Nevertheless, the conditions for in operando observation still have two major factors different from the real SSLB. 1) High vacuum environment. For the real SSLB, the vacuum environment of in operando TEM sample is higher which can further induce failure behavior between the LCO/LLZO interface. These relevant results will be described in the following information. 2) Thickness of the sample. In order to observe the microstructural variation of SSLB and the reaction between the interfaces, the thickness of the specimen observed via in operando TEM is much thinner than that of bulk‐SSLB. Due to the extremely thin sample, it is slightly different from bulk‐SSLB in resistance and electrochemical potential. Furthermore, the very thin TEM sample gives a very small amount of extractable Li‐ions which presents a technical challenge in controlling current density instead of voltage during electrochemical cycling under TEM. Nevertheless, we can still adjust the parameters of the in operando observation to simulate the operation of the real SSLB, the corresponding information will be discussed in detail. First, the SSLB sample was charged to 3 V versus open circuit voltage (OCV). However, the sample was melted due to the domination of Joule heat, which indicates the voltage was too high. In consideration of the LCO thin slice being tiny, with a dimension of ≈100 nm in thickness and 1 to 2 µm in width and length, for example, Figure S1c, Supporting Information, we suspected that the amount of Li can be taken out from the LCO thin slice is not enough to alloy with Pt electrode to give a correct electrochemical potential of the battery. When considering the OCV of the as‐assembled bulk‐type SSLB, the OCVs for the batteries using Li anode usually have OCV ≈2.75 V while that for using Pt anodes could vary from 1 to 0.15 V (Figure S2, Supporting Information). Furthermore, the reported OCV for the as‐prepared batteries by using Pt as the anode also gave OCV around 2.7 V versus Li/Li^+^.^[^ ^82^, ^83^ ^]^ Therefore, another SSLB prepared by FIB for TEM investigation was conducted by using a charging rate of 0.05 V s^−1^ to reach 1.5 V versus OCV and then kept the voltage at 1.5 V for 10 min. During discharging, a negative bias was applied at a rate of 0.05 V s^−1^ to reach −0.5 V versus OCV and then also keep 10 min at −0.5 V. The charge/discharge cycle was repeated ten times for the whole investigation as shown in Figure S3, Supporting Information. With this strategy, we are hoping that the charging potential could reach ≈4.2 V versus Li/Li^+^, and the discharge voltage would be ≈2.2 V versus Li/Li^+^.

**Figure 2:**
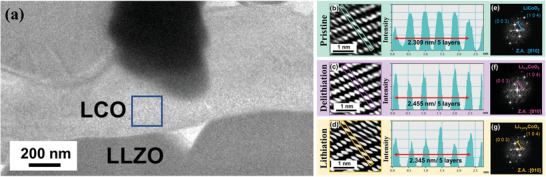
TEM and HRTEM images and electron diffraction patterns show the difference in d‐spacing after delithiation/lithiation of the SSLB. a) TEM image of the pristine sample, b–d) the corresponding HRTEM image for the pristine, delithiated, and lithiated samples, and e–g) the corresponding electron diffraction pattern for the pristine, delithiated, and lithiated samples.

The last 80 s of 1st delithiation and lithiation process observed via in operando TEM are presented in Movies S1 and S2, Supporting Information, respectively, whose speeds were adjusted to four times faster than actually were. The microstructure analysis after delithiation/lithiation of the SSLB is demonstrated in **Figure** **2**. Additionally, after the 1st cycle, the interface between LLZO and LCO displayed remains clean with no obvious change; the corresponding TEM image is shown in Figure 2a. The EDS elemental mappings for the pristine sample and after the 1st delithiation and lithiation are shown in Figure S4, Supporting Information. In Figure S4, Supporting Information, the signals of cobalt (Co) can be used to represent the LCO phase while lanthanum (La) and zirconium (Zr) can be used to represent the LLZO phase. It should be noted that the Zr signals were spread into the LCO phase at the lower part of the EDS mapping. When comparing with the EDS oxygen mapping, it can be concluded that the spread of Zr into the LCO phase can be associated with the Pt electrode because the Zr_L*α* line (2.042 keV) and Pt_M line (2.048 keV) have very similar characteristic X‐ray energies, as well as the Zr signal is highly overlapping with Pt positions in the LCO grain. When focusing on the investigated interface between LLZO and LCO, these EDS elemental mappings show that there was no obvious elemental interdiffusion caused by the electrochemical delithiation/lithiation of the SSLB. Although there is no recognizable morphology change of LCO after 1st electrochemical cycle, the atomic‐scale TEM images provide strong evidence for the delithiation/lithiation process from the change of LCO d‐spacing. The atomic‐resolution TEM image of pristine LCO is shown in Figure 2b, where the average d‐spacing of LCO_(003)_ is 0.462 nm by averaging from the five layers of CoO_6_‐Li‐CoO_6_. The corresponding electron diffraction pattern with a zone axis of [010] is shown in Figure 2e. This is in agreement with previous reports.^[^ ^86^, ^87^, ^88^, ^89^, ^90^ ^]^ During the delithiation process, Li‐ions were extracted from LCO. The Li vacancies generated by the delithiation process change the original CoO_6_‐Li‐CoO_6_ to CoO_6_‐□‐CoO_6_ (□: Li vacancy), which increases the Coulomb repulsion between CoO_6_, and hence, the d‐spacing of Li_1‐_ *_x_* CoO_2_ increases.^[^ ^90^, ^91^, ^92^ ^]^ Therefore, the d‐spacing of Li_1‐_ *_x_* CoO_2 (003)_ increases by 5.9% from 0.462 to 0.491 nm (averaged from five layers of CoO_6_‐Li/□‐CoO_6_), as indicated in Figure 2c, resulting in an electron diffraction pattern with a zone axis of [010] in Figure 2f. The successful delithiation of LCO is also supported by electron energy loss spectroscopy (EELS) (Figure S5, Supporting Information). The L‐edge spectra of Co shift to a higher energy level from the pristine one indicating that the valence state of Co‐ions changes from +3 to +3/+4. However, the measured d‐spacing of Li_1‐_ *_x_* CoO_2 (003)_ is a little bit higher than the previous scanning transmission electron microscopy (STEM) reports, it could be a result of the Joule heating during the measurement, especially with the 10 min constant voltage duration.^[^ ^80^, ^91^ ^]^ In the following lithiation process, Li‐ions diffuse back to the Li vacancies in Li_1‐_ *_x_* CoO_2_. The Coulomb repulsion between CoO_6_ was reduced, which leads the d‐spacing of Li_1‐_ *_x_*_+_ *_y_* CoO_2 (003)_ reducing from 0.491 to 0.469 nm. The HRTEM image after lithiation and the corresponding electron diffraction pattern (with a zone axis of [010]) are shown in Figures 1d and 1g, respectively. The increase of the d‐spacing from pristine LCO (0.462 nm) to lithiated LCO (0.469 nm) indicates that trace amounts of Li‐ions did not return to their original sites of LCO. This could be a result of some Li atoms being trapped in the Pt anode. Nevertheless, the d‐spacing of lithiated Li_1‐_ *_x_*_+_ *_y_* CoO_2 (003)_ is in agreement with the reported d‐spacing of Li_1‐_ *_x_*_+_ *_y_* CoO_2 (003)_ that was measured by TEM after cycling.^[^ ^92^, ^93^, ^94^ ^]^

**Figure 3:**
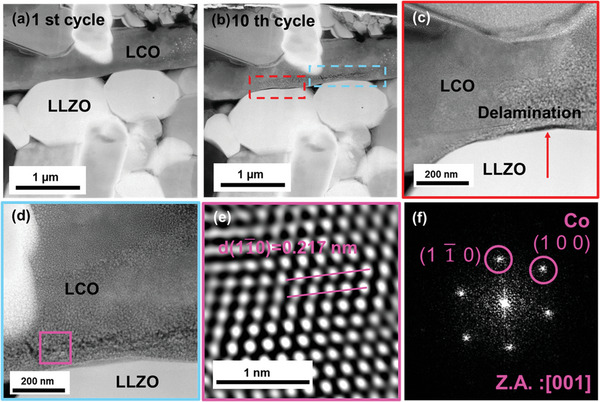
SSLB capacity degradation analysis after electrochemical cycles. STEM images show the morphology of the SSLB after a) 1st cycle and b) 10th electrochemical cycle. LLZO/LCO interfacial delamination and a microcrack formation within LCO were observed. STEM images of c) interfacial delamination and d) microcrack formation within LCO after ten electrochemical cycles. e) HRTEM image shows the Co precipitation near the microcrack of LCO and f) its corresponding electron diffraction pattern.

According to the evolution of the d‐spacing of LCO_(003)_ upon delithiation, the d‐spacing of LCO_(003)_ should be at the maximum when it is delithiated to ≈Li_0.5_CoO_2_.^[^ ^80^, ^93^, ^94^, ^95^ ^]^ Further delithiation or lithiation only decreases the d‐spacing of LCO_(003)_. Therefore, we believe that our strategy for electrochemically delithiation of the SSLB under TEM to a voltage of ≈4.2 V versus Li/Li^+^ and the following lithiation of the SSLB sample under TEM were successful. Therefore, another nine electrochemical cycles were conducted under the same profile for degradation mechanism investigation. The degradation behaviors are displayed in **Figure** **3**. Figure 3a shows the STEM image of the SSLB after the 1st electrochemical cycle, for which the interface between LLZO and LCO maintains good contact. However, delamination was observed on the LLZO/LCO interface, as well as a microcrack was found within the LCO grain after ten electrochemical cycles. The STEM image for the SSLB after the 10th electrochemical cycle is shown in Figure 3b, where the red dotted frame indicates the delamination between LLZO and LCO on the interface. The enlarged STEM image of the red dotted frame area is shown in Figure 3c. During electrochemical cycling, the change in Li molar volume leads to the LCO volume expanding/contracting. Compressive/tensile stresses evolvement on the LLZO/LCO interface leads to the delamination and the afterward crack evolution. Barai et al. point out in their model that the interface of LLZO/LCO should have minor changes during the delithiation process due to the generation of compressive stress on the interface from the increase of LCO volume.^[^ ^72^ ^]^ The delamination on the interface of LLZO/LCO should be majorly due to the tensile stress during the following lithiation process. Nevertheless, the step‐like discharge curve was not observed in our SSLB, Figure 1e, which suggests that the delamination of the LLZO/LCO interface should be developed in a slow process.

In addition, the light blue dotted frame in Figure 3b reveals a formation of microcracks within the LCO grain, whose enlarged STEM image is shown in Figure 3d. It is reported that layered transition metal oxides, such as LiNi_0.8_Co_0.15_Al_0.05_O_2_ and LCO, form oxygen vacancies when heat treated at a temperature higher than 400 and 600 °C, respectively.^[^ ^96^, ^97^ ^]^ This is due to the lattice oxygen attempt to reach the equilibrium of the chemical potential with environmental oxygen by the thermodynamic driving force. Lattice oxygen vacancies are energetically favored once the chemical potential for the environment oxygen is lower at high temperatures. Considering the sintering temperature of the CPE was 970 °C in air, the formation of oxygen vacancies within the LCO structure is highly conceivable. The presence of oxygen vacancies in LCO not only causes large local strain that initiates the microcracking within LCO but also facilitates the migration of Co to escape from the host lattice to form metallic Co and release oxygen during electrochemical cycling.^[^ ^96^, ^98^, ^99^ ^]^ This is in agreement with our observation that metallic Co was detected near the microcrack, for which the HRTEM image is shown in Figure 3e. The corresponding d‐spacing is 0.217 nm for ${\mathrm{Co}}_{\left(1\bar{1}0\right)}$ and its electron diffraction pattern with a zone axis of [001] is shown in Figure 3f. It is, therefore, identified the precipitations are metallic Co with space group P63/mmc.^[^ ^100^ ^]^ In addition, the release of oxygen would further lead to the growth and propagation of the microcrack within or even throughout the LCO grains. Interface behavior at a higher delithiation state by applying 2 V (i.e., ≈4.7 V vs Li/Li^+^) to a specimen was also studied by in operando TEM. The results are shown in Movie S3 and Figure S6, Supporting Information. The delamination of the LCO/LLZO interface during the delithiation process can be clearly seen in the movie. This is because the higher volume change of LCO from the hexagonal phase to the high voltage monoclinic phase or even the CoO_2_ phase causes high tensile stress to break the interface immediately.

**Figure 4:**
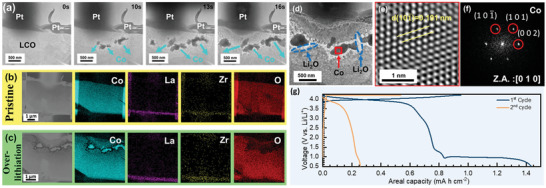
Structural identification and analysis of the over‐lithiated SSLB. a) TEM images of time‐dependent over‐lithiation process on an LCO particle. Metallic Co is gradually precipitated in LCO. EDS elemental mappings of b) pristine sample and c) after over‐lithiation, where Co represents the LCO phase and La and Zr represent the LLZO phase. d) Low magnification TEM image on LCO after the over‐lithiation of the SSLB. e) HRTEM image of the precipitated Co which is marked by the red frame in (d) and f) its corresponding electron diffraction pattern. g) Electrochemical cycle of a bulk‐type SSLB down to 0.5 V versus Li/Li^+^ for over‐lithiation study.

In addition, an ultralow working voltage of −1.5 V (i.e., ≈1.2 V vs Li/Li^+^) was applied to another specimen where another degradation mechanism of the SSLB was observed. For better observation of the degradation mechanism under TEM, a layered type SSLB, where the upper layer was LCO and the lower one was LLZO, was used for the investigation. **Figure** **4**a and Movie S4, Supporting Information, reveal the over‐lithiation process at the ultralow voltage, where agglomerates were observed to precipitate within the LCO grain. The corresponding EDS elemental mappings of the pristine and over‐lithiated SSLB are shown in Figures 4b and 4c, respectively. It can be clearly seen that the agglomerates are composed of highly concentrated Co, for which ≈95 at% of the material was identified by EDS point analysis to be Co and ≈5 at% to be oxygen (Figure S7, Supporting Information). Furthermore, TEM was also used to investigate the agglomerates after the over‐lithiation process (Figure 4d), where the red frame was selected to analyze the atomic structure of the precipitation by HRTEM (Figure 4e). The corresponding d‐spacing is 0.191 nm for $Co_{\left(10\bar{1}\right)}$ and its electron diffraction pattern with a zone axis of [010] is shown in Figure 4f. It is, therefore, identified that the precipitations are also metallic Co with space group P6_3_/mmc.^[^ ^100^ ^]^ Furthermore, a polycrystalline Li_2_O electron diffraction ring was obtained next to the metallic Co agglomerates, Figure S8, Supporting Information. This indicates that LCO was decomposed into metallic Co and Li_2_O during the over‐lithiation process. The same experiment was also carried out by using a specimen prepared from CPE (Figure S10, Supporting Information). Its EDS elemental mappings and point analysis also reveal the same degradation mechanism as that for the layered type SSLB (Figures S9 and S10, Supporting Information).

From the previous studies on LCO over‐lithiation mechanism by using organic liquid electrolyte and metallic Li as the anode, Shu et al. suggested that the over‐lithiation process of LCO from 4.3 to 1 V versus Li/Li^+^ can be described as

(1) $$\begin{array}{ccc} & & {\mathrm{Li}}_{x}{\mathrm{CoO}}_{y}+\left(6y-3x-8\right){\mathrm{Li}}^{+}+\left(6y-3x-8\right)e^{-}↔{\mathrm{Co}}_{3}O_{4}\\ & & \;+\;\left(3y-4\right){\mathrm{Li}}_{2}O \end{array}$$

(2) $${\mathrm{Co}}_{3}O_{4}+2{\mathrm{Li}}^{+}+2e^{-}↔3\mathrm{CoO}+{\mathrm{Li}}_{2}O$$

(3) $$\mathrm{CoO}+2{\mathrm{Li}}^{+}+2e^{-}↔{\mathrm{Li}}_{2}O+\mathrm{Co}$$

where Li_*x*_CoO_*y*_ (1.34 + 0.5*x* ≤ *y* ≤ 1.5 + 0.5*x*). Nevertheless, the system that studied by in operando TEM was using Pt as the anode, which means that the explanation would not fit this scenario due to the lack of Li source from the anode (Pt). A bulk‐type LLZO‐based SSLB was also subjected to a cycle in the voltage between 4.2 and 0.5 V versus Li/Li^+^ (Figure 4g). For the 1st discharge cycle of the SSLB, the discharge behavior is rather similar to the 2nd discharge cycle from the reports of Shu et al.^[^ ^89^ ^]^ and Yu et al.,^[^ ^79^ ^]^ where three distinguishable plateaus can be observed, that is, ≈3.97, ≈1.25, and ≈0.96 V versus Li/Li^+^. The plateau at ≈3.97 V versus Li/Li^+^ can be assigned to the typical LCO discharge behavior when cycling between 4.2 and 3.4 V versus Li/Li^+^, as in Figure 1e. A plateau at ≈1.25 V versus Li/Li^+^ is assigned to the reaction of LCO and Li‐ions to form Co_3_O_4_ and Li_2_O, that is, Equation (1), while the plateau at ≈0.96 V versus Li/Li^+^ is the reaction of cobalt oxides (Co_3_O_4_ and CoO) and Li‐ions to form Li_2_O and metallic Co, that is, Equations (2) and (3), according to Shu et al.^[^ ^89^ ^]^ It is also noticed that the specific capacity of LCO is much lower for the SSLB (232 mA h g^−1^ to 0.5 V vs Li/Li^+^) than that for using liquid electrolyte cells (≈1000 mA h g^−1^), where the plateau at ≈0.96 V versus Li/Li^+^ only delivers 96 mA h g^−1^ for the SSLB while that for liquid electrolyte cells is about 800 mA h g^−1^.^[^ ^79^, ^89^, ^101^ ^]^ Furthermore, the discharge plateaus at ≈1.25 and ≈0.96 V versus Li/Li^+^ were no more observed in the 2nd discharge cycle. Unlike liquid electrolytes which can penetrate through the newly formed materials to provide Li‐ion conductive paths, the much lower specific capacity and the disappearing of the two discharge plateaus at ≈1.25 and ≈0.96 V versus Li/Li^+^ can be explained by the reduction of Li‐ion conductive paths through LCO after LCO was decomposed into cobalt oxides and Li_2_O. Also, the very small specific capacity for the plateau ≈1.25 V versus Li/Li^+^ implies that LCO could decompose into metallic Co and Li_2_O directly through a metastable phase Li_1 + *x*_Co^II III^O_2 − *y*_ (0 < *x*, 0 ≤ y), as suggested by Shu et al.^[^ ^89^ ^]^ The loss of Li‐ion conductive paths through LCO also led to the high polarization of the cell for the 2nd charging process. This results in the capacity retention of the 2nd discharge being only 25% of that for the 1st discharge cycle when calculating the discharge‐specific capacity of LCO at 3.4 V versus Li/Li^+^.

Combine with the bulk‐type SSLB over‐lithiation behavior and the in operando TEM results, the over‐lithiation study in in operando TEM suggests that LCO should be intrinsically not stable under low voltage bias, which leads to the decomposition of LCO into metallic Co, cobalt oxides, and Li_2_O. Cobalt oxides could further decompose into metallic Co and release oxygen due to the ultra‐low pressure TEM environment and Joule heat from the electron beam of TEM.^[^ ^102^ ^]^ However, the formation of cobalt oxide was not observed in the in operando TEM. This is similar to the report from in operando X‐ray absorption near‐edge spectroscopy and in operando transmission X‐ray microscopy.^[^ ^101^ ^]^ It further suggests that most of the LCO would decompose directly into metallic Co and Li_2_O when considering the low capacity at the plateau ≈1.25 V versus Li/Li^+^.

## 3 Discussion

**Figure 5:**
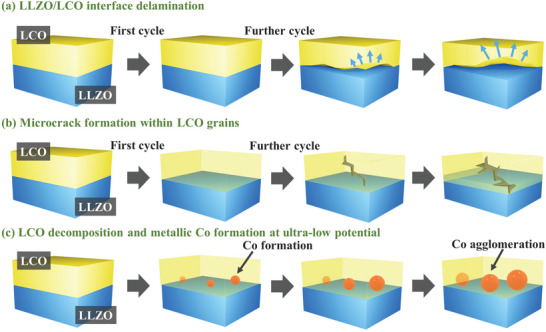
Schematic of the SSLB capacity degradation mechanisms during electrochemical cycling. a) LLZO/LCO interface delamination during the electrochemical cycling owing to the volume change of LCO. b) Microcrack formation within LCO owing to oxygen vacancy‐induced high local stress. c) LCO decomposition into metallic Co and Li_2_O when subjected to an over‐lithiation to ≈1.2 V versus Li/Li^+^.

By using in operando TEM, the delithiation/lithiation of SSLB was successfully demonstrated and investigated. The schematic illustration for the degradation mechanism of SSLB is indicated in **Figure** **5**. During delithiation, the extraction of Li ions causes the increase of Coulomb repulsion between CoO_6_‐□‐CoO_6_ that leads to the increase of the d‐spacing of Li_0.5_CoO_2_. In contrast, the d‐spacing of Li_0.5_CoO_2_ decrease after the lithiation because Li ions returned to the initial sites of LCO. In addition, the capacity degradation mechanisms of the SSLB were investigated. During the normal cycling between 4.2 and 2.2 V versus Li/Li^+^, the repeated volume change of LCO due to the delithiation/lithiation process leads to compressive/tensile stresses evolvement on the LLZO/LCO interface, which leads to the delamination of the LLZO/LCO interface, Figure 5a. Furthermore, the development of microcracks within the LCO grain was also observed, Figure 5b. This degradation mechanism is triggered by the presence of oxygen vacancies in LCO grains due to the high sintering temperature of CPE. During electrochemical cycling, the oxygen vacancies in LCO cause high local strain to initiate the microcrack and further lead to metallic Co formation and oxygen release from LCO. Both the interfacial delamination and microcrack formation in LCO grains result in higher tortuosity for Li‐ion and electronic diffusion, which increases the total resistance of the battery. As the result, the charge/discharge voltage would hit the cut‐off voltage at a lower capacity when a fixed charge/discharge current density is applied. In addition, the LCO decomposition initiated by the presence of oxygen vacancies within LCO grains is further contributing to the capacity degradation of the SSLB. Although the volume change of LCO during electrochemical cycling is considered low, < 3% when cycling Li*_x_* CoO_2_ for *x* between 1 and 0.5, the impact from this small volume change is still significant. Therefore, the application of zero‐strain cathode materials would be one of the choices to eliminate the impact of stress development during electrochemical cycling.^[^ ^103^, ^104^, ^105^, ^106^ ^]^ It is also known that the elastic modulus is a function of ceramic's microstructure, a further optimization of the CPE microstructure for higher stress tolerance to avoid interface delamination may still be feasible.^[^ ^107^, ^108^ ^]^ Furthermore, changing the SSLB sintering environment from air to dry pure oxygen atmosphere would be able to reduce the impurity such as Li_2_CO_3_ formation as well as reduced oxygen vacancy formation of LCO to avoid the microcrack formation within its grains. When the SSLB is over‐lithiated to a voltage of 1.2 V versus Li/Li^+^, LCO decomposes into metallic Co and Li_2_O, Figure 5c. The decomposition of LCO further cut off the Li‐ion diffusion paths in the SSLB to access the newly formed metallic Co and Li_2_O, which results in a very high irreversible capacity loss when the SSLB is over‐lithiated. Therefore, the electrochemical cycling of SSLB with LCO as the cathode should stay at a voltage above 1.5 V versus Li/Li^+^ or preferably higher than 3 V versus Li/Li^+^ to avoid LCO decomposition.

In summary, LLZO‐based SSLB was prepared with optimized sintering conditions to show clear LCO electrochemical behaviors. The EDS elemental mappings and XRD results also demonstrate that the LCO phase was maintained after the high‐temperature sintering process. The fabricated SSLB was able to be cycled with a high areal current density at 50 µA cm^−2^, to give a high discharge areal capacity of 0.63 mA h cm^−2^ and to demonstrate a high capacity retention of 80.8% after 100 cycles. Delithiation/lithiation of LCO under in operando TEM was successfully demonstrated by monitoring the change of the d‐spacing of LCO. Detailed investigation of the LLZO/LCO interface reveals that stress evolvement from the delithiation/lithiation of LCO causes the delamination of the interface. Furthermore, the oxygen vacancy formation within LCO from the high‐temperature sintering process causes high local strain to trigger the microcrack in LCO. Both of them contribute to the degradation of SSLB during normal electrochemical cycling. When cycling the SSLB down to 1.2 V versus Li/Li^+^, LCO decomposes into metallic Co and Li_2_O. The interdict of Li‐ion diffusion paths within CPE to re‐access the metallic Co and Li_2_O leads to very high irreversible capacity fading. The results suggest that further optimization of the microstructure of CPE for higher stress tolerance and processing the LLSB under a pure oxygen environment would further improve the SSLB electrochemical cycling performance when using LCO as the AC. Furthermore, the SSLB should not lithiate to a voltage below 1.5 V versus Li/Li^+^ to avoid LCO irreversible decomposition.

## 4 Experimental Section

### Fabrication of SSLB

As for the experimental section, the fabrication of Li_6.45_Al_0.05_La_3_Zr_1.6_Ta_0.4_O_12_ (LLZTaO) and the SSLB followed the previous publication.^[^ ^18^ ^]^ All chemicals used in this publication were the same as the previous one. Here, only a brief description and the change of parameters from the previous publication are provided.

Stoichiometric amounts of the starting materials, LiOH∙H_2_O (Merck, 98%), La_2_O_3_ (Merck, 99.9%, pre‐dried at 900 °C for 10 h), ZrO_2_ (Treibacher, 99.5%), Ta2O5 (Inframat, 99.95%), and *α*‐Al_2_O_3_ (Inframat, 99.9%) were dry mixed for the calcination processes. The calcination processes comprised three‐step calcinations, once at 850 °C and twice at 1175 °C, with a dwell time of 20 h. Grinding and pressing were repeated between the calcination steps. The LLZTaO pellets were sintered at 1175 °C for 10 h. After the sintering step, the LLZTaO pellets were sliced into the desired discs with a thickness of ≈0.6 mm.

The SSLB was fabricated by sintering CPE onto LLZTaO discs. The CPE was prepared using a 1:1 mass ratio of LCO (MTI Corp., USA), 5.06 g cm^−3^, and LLZTaO, 5.35 g cm^−3^, powder. A screen printing ink of CPE was prepared by mixing 3 wt% ethyl cellulose (Sigma‐Aldrich) in terpineol (Sigma‐Aldrich) and CPE solid loading with a weight ratio of 1:1 using three‐roll milling. Afterward, the ink was painted onto the LLZTaO discs with a brush. After drying, the half‐cells were sintered in a tube furnace (Nabertherm, Germany) at 970 °C for 15 min with a heating rate of 6.5 °C min^−1^ and free cooling in air.

After the half‐cell sintering, samples were polished on the LLZTaO side to remove possible impurities and thin the solid electrolyte down to ≈400 µm by using SiC sandpaper. An Au‐thin film was sputtered on the top of the CPE to serve as a current collector. Then, the batteries were assembled using Li foil as the anode and heated up to 220 °C for 5 min to reduce the interface resistance between Li and LLZTaO on a hot plate before being put into Swagelok cells for electrochemical measurements. Within the Swagelok cell, a spring with a spring constant of 10 N cm^−1^ was used for providing proper electric contact and pressure of ≈0.1 MPa to the battery. The battery assembly was done in a high‐purity Ar‐filled glove box. The CPE loading was 12 mg cm^−2^ for the SSLB that underwent electrochemical cycling as shown in Figure 1d,e, which gave an LCO loading of 6 mg cm^−2^. Moreover, SEM images of half of SSLB microstructure from large scale down to the interface of CPE/LLZO are shown in Figure S11, Supporting Information, to demonstrate the well sintering of CPE onto LLZO.

### Performance measurement and characterization

The electrochemical performance of the fabricated SSLB was carried out at 60 °C by using a BioLogic VMP‐300 multipotentiostat combined with a climate chamber (Binder, Germany). The cycling of the battery was done by using a constant‐current‐constant‐voltage process. The battery was charged to 4.2 V versus Li/Li^+^ with a constant current density of 50 µA cm^−2^ and then held at 4.2 V versus Li/Li^+^ until the current dropped to 10 µA cm^−2^. Then, the discharge was conducted with a constant current density of 50 µA cm^−2^ until the voltage dropped to 3.4 V versus Li/Li^+^. No other additive, liquid, or polymer electrolyte was involved in the fabricated SSLB.

The microstructures and elemental chemistry distribution of CPE were investigated by using a Quanta 650 FEG scanning electron microscope (SEM) (FEI Company, USA) equipped with an EDS (Ametek, USA).

### In operando TEM sample preparation and observation

The samples for in operando TEM observations were prepared using a FIB system (TESCAN GAIA3) from the CPE. To avoid radiation damage from Ga ions, a platinum 0(Pt) layer was deposited on the surface of the SSLB (width 12 µm, height 1.5 µm, thickness 1.5 µm) as the protection layer, as shown in Figure S12a, Supporting Information. Then, trenches whose width and depth were 15 and 6 µm, respectively, were milled on both sides of the platinum to provide sufficient space for cutting (Figure S12b, Supporting Information). Rough polishing was introduced to remove impurities and contaminants, as shown in Figure S12c, Supporting Information. Furthermore, the sample was hung on the bulk because the left and bottom of the sample were cut by U‐cutting (Figure S12d, Supporting Information). Subsequently, the thickness of the sample was controlled below 100 nm (Figure S12e, Supporting Information) for better investigation of the HRTEM images, followed by lifting the sample out by cutting, as shown in Figure S12f, Supporting Information. Pt wires were deposited by FIB to connect the electrolyte and LCO electrode of the sample with the chip electrode after transferring the sample onto an in operando TEM chip using a glass tip (Figure S13a,b, Supporting Information). Subsequently, the chip was loaded into the in operando TEM holder, as shown in Figure S14c, Supporting Information (Protochips Audro300). The dynamic cycling process was recorded using a JEOL F200 equipped with a OneView camera and an EDS. Further, XRD was conducted to identify the crystal structure of the initial samples. XRD was carried out by using an EMPYREAN (Panalytical) with Ni‐filtered Cu K*α* radiation. A sample for XRD was collected from the sintered CPE. The diffractograms were collected in a 2*θ* range from 10° to 80° at 40 kV, 40 mA, with a step size of 0.008° 2*θ*, and a counting time of 20 s per step. In addition, Cs‐corrected STEM (JEOL ARM 200F) was conducted to identify the valence states of the SSLB constituent elements at various charge states.

## Conflict of Interest

The authors declare no conflict of interest.

## Author Contributions

All authors made their major contributions to this research project and manuscript preparation. All authors also have given their final approval for the publication.

## Supporting information

Supporting Information

Supplemental Movie 1

Supplemental Movie 2

Supplemental Movie 3

Supplemental Movie 4

## Data Availability

The data that support the findings of this study are available from the corresponding author upon reasonable request.
