# Supplementary material for: All‐Solid‐State Garnet‐Based Lithium Batteries at Work–In Operando TEM Investigations of Delithiation/Lithiation Process and Capacity Degradation Mechanism
Source: Adv Sci (Weinh). 2022 Dec 18;10(5):2205012. doi: 10.1002/advs.202205012 (PMC9929109; doi:10.1002/advs.202205012)
Supplement: Supplementary file 1 — Supporting Information [file ADVS-10-2205012-s003.pdf]

ADVANCED SCIENCE
Open Access

Supporting Information

for *Adv. Sci.*, DOI 10.1002/advs.202205012

All-Solid-State Garnet-Based Lithium Batteries at Work–In Operando TEM Investigations of Delithiation/Lithiation Process and Capacity Degradation Mechanism

An-Yuan Hou, Chih-Yang Huang, Chih-Long Tsai, Chun-Wei Huang, Roland Schierholz, Hung-Yang Lo, Hermann Tempel, Hans Kungl, Rüdiger-A. Eichel, Jeng-Kuei Chang and Wen-Wei Wu**

Supporting Information

All Solid-State Garnet-based Lithium Batteries at Work – In Operando TEM Investigations of Delithiation/Lithiation Process and Capacity Degradation Mechanism

*An-Yuan Hou, Chih-Yang Huang, Chih-Long Tsai, Chun-Wei Huang, Roland Schierholz , Hung-Yang Lo , Hermann Tempel , Hans Kungl , Rüdiger-A. Eiche**, Jeng-Kuei Chang and Wen-Wei Wu**

List of movie Contents.

Movie S1. *In operando* TEM video of the last 80 seconds of the 1st delithiation process. No clear change of the microstructure of the SSLB was observed. The video is playing at four times of the actual time speed.

Movie S2. *In operando* TEM video of the last 80 seconds of 1st lithiation process. No clear change of the microstructure of the SSLB was observed. The video is playing at four times of the actual time speed.

Movie S3. *In operando* TEM video shows LLZO/LCO interface delamination when the SSLB subjects to delithiate to 2 V vs. OCV (i.e. ~ 4.7 V vs. Li/Li^+). The delamination was observed in one delithiation process.

Movie S4. *In operando* TEM video demonstrates Co precipitations during the over-lithiation process. LCO decomposes into metallic Co and Li_2O when applies a working voltage of -1.5 V (i.e. ~1.2 V vs Li/Li^+).

Figure S1. LLZO-based all-solid-state Li battery structural analysis of the pristine sample.

Figure S2. Open circuit voltage (OCV) of the as prepared SSLB (Bulk type) with Pt or Li anode.

Figure S3. Electrochemical cycling profile for the multiple cycling process by *in operando* TEM investigation.

Figure S4. Low magnification of STEM images, EDS mapping for pristine, delithiated, and lithiated sample.

Figure S5. EELS analysis of the SSLB.

Figure S6. STEM images of a SSLB subjects to delithiate to 2 V vs. OCV (i.e. ~ 4.7 V vs. Li/Li^+).

Figure S7. EDS point analyses of the precipitations.

Figure S8. Electron diffraction pattern of polycrystalline Li_2O, which found next to the precipitated Co after the over-lithiation.

Figure S9. EDS elemental mappings of the pristine and after over-lithiated SSLB sample.

Figure S10. EDS point analyses of the over-lithiated CPE sample.
Figure S11. SEM images of SSLB after sintering of CPE onto LLZO.
Figure S12. Sample preparation of SSLB by FIB for the *in operando* TEM investigation.
Figure S13. Pt wires deposited by FIB.

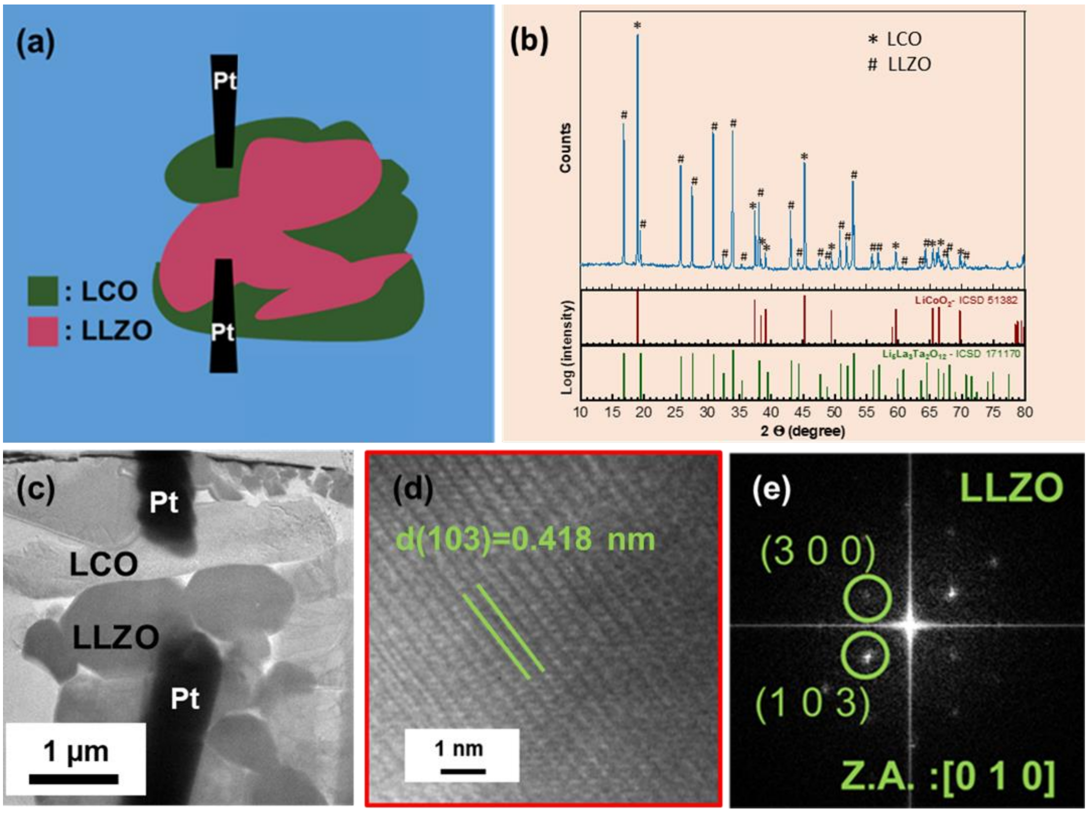

Figure S1. LLZO-based all-solid-state Li battery structural analysis of the pristine sample. (a) schematic of prepared SSLB for TEM analysis. (b) XRD diffraction pattern of sintered CPE. Only LLZO and LCO were detected after the sintering process. (c) low magnification TEM image of the SSLB for TEM investigation. (d) HRTEM images of the LLZO and (e) its corresponding electron diffraction patterns.

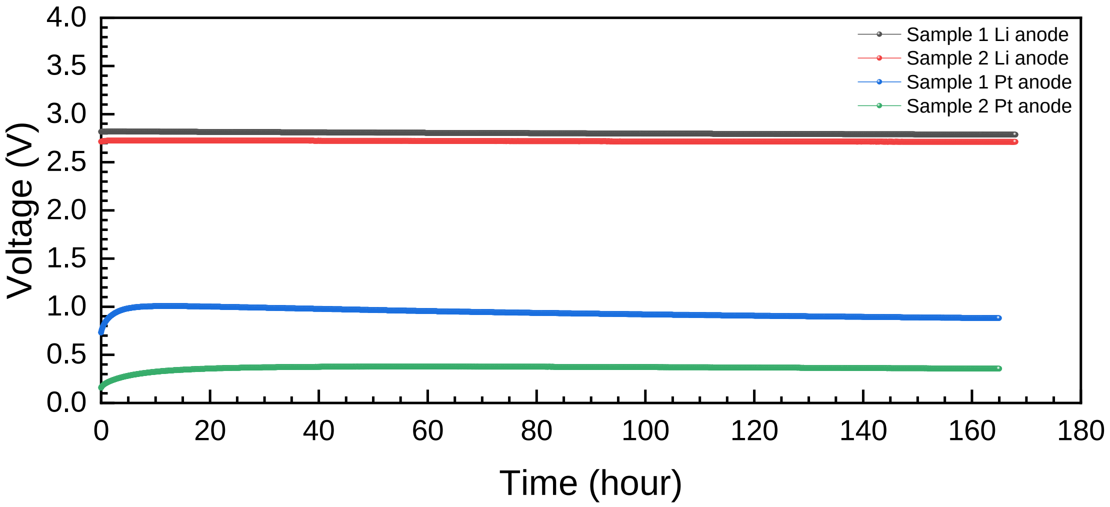

Figure S2. Open circuit voltage (OCV) of the as prepared SSLB (Bulk type) with Pt or Li anode. SSLBs using Li as the anode usually have OCVs ~ 2.75 V while that for Pt anodes can change from 0.35 V to 0.88 V.

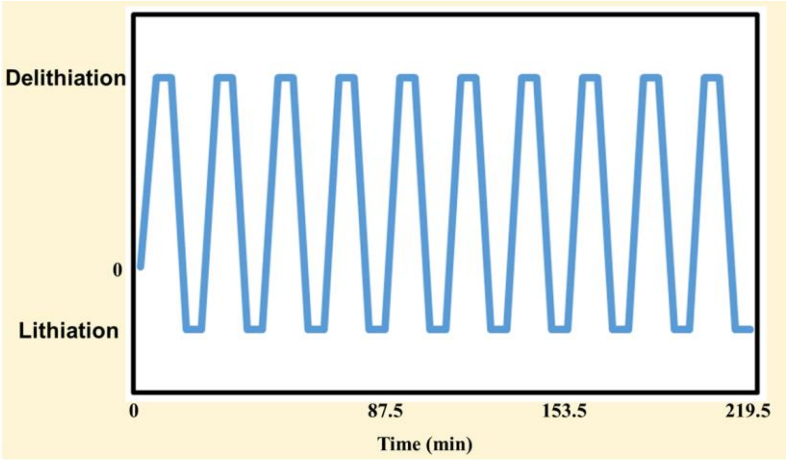

Figure S3. Electrochemical cycling profile for the multiple cycling process by *in operando* TEM investigation. The charge rate was set to 0.05 V/s to reach 1.5 V vs. OCV and maintained for 10 minutes. The discharge rate was also set to 0.05 V/s to reach -0.5 V vs. OCV and maintained for 10 minutes, The entire investigation was repeated ten times.

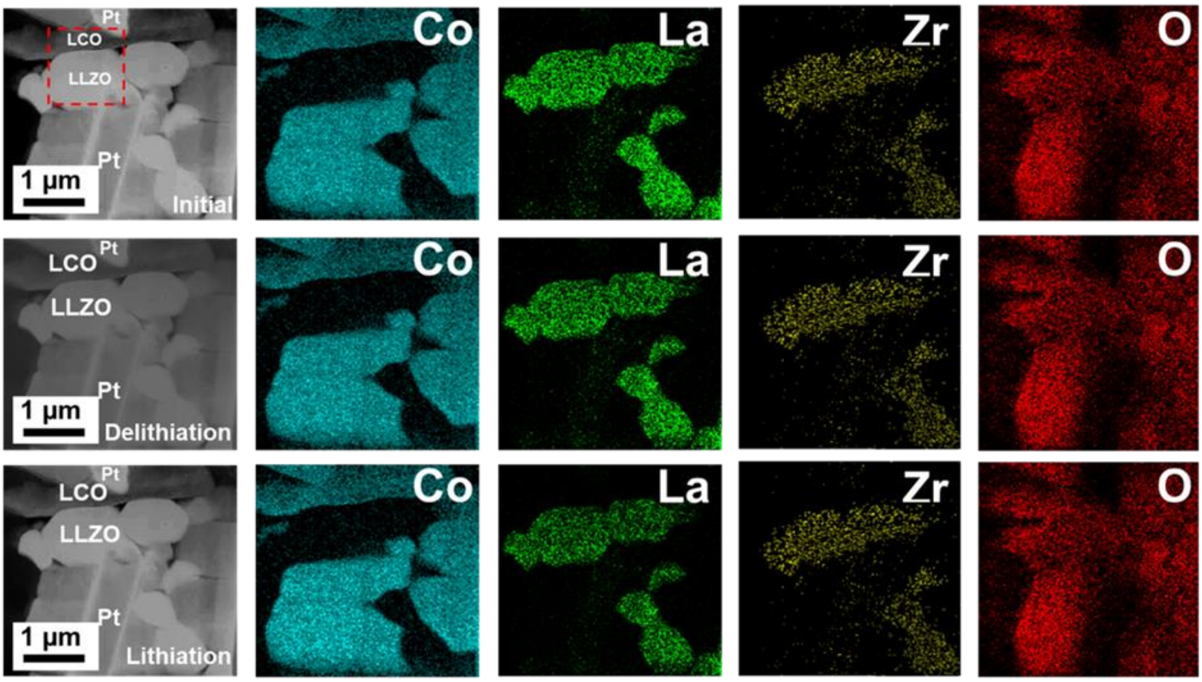

Figure S4. Low magnification of STEM images, EDS mapping for pristine, delithiated, and lithiated sample. The distribution of Co indicates the LCO, while the region of LLZO represented by La and Zr elements.

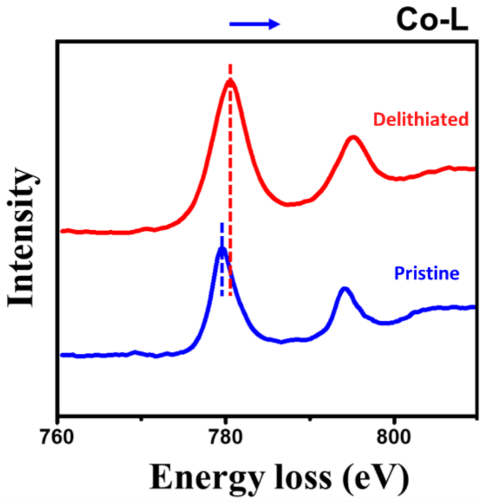

Figure S5. EELS analysis of the SSLB. EELS results of the L-edge spectra of Co showing that the delithiated Co-ions shifts to higher energy levels, which indicates the valence state of Co-ions were increase from Co^{III} to $Co^{III/IV}$.

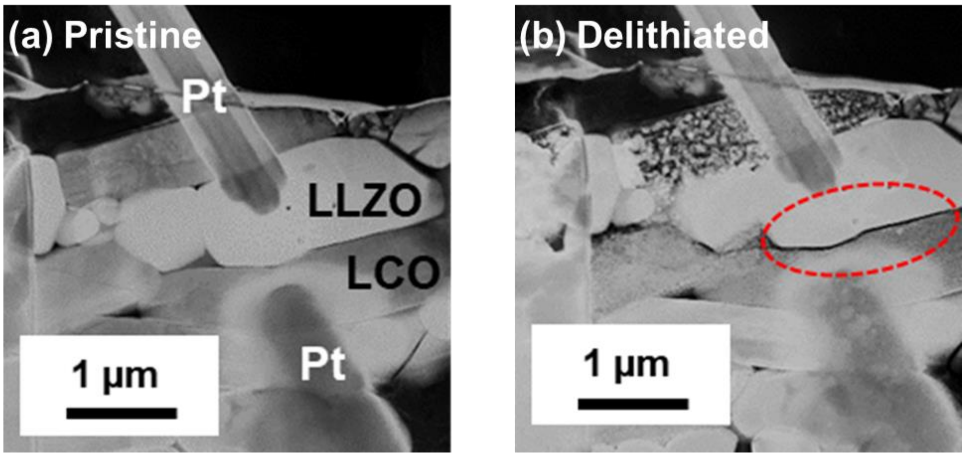

Figure S6. STEM images of a SSLB subjects to delithiate to 2 V vs. OCV (i.e. ~ 4.7 V vs. Li/Li^+). STEM images of (a) pristine, and (b) delithiated samples. LLZO/LCO interface delamination can be seen immediately (red dot circle in (b)).

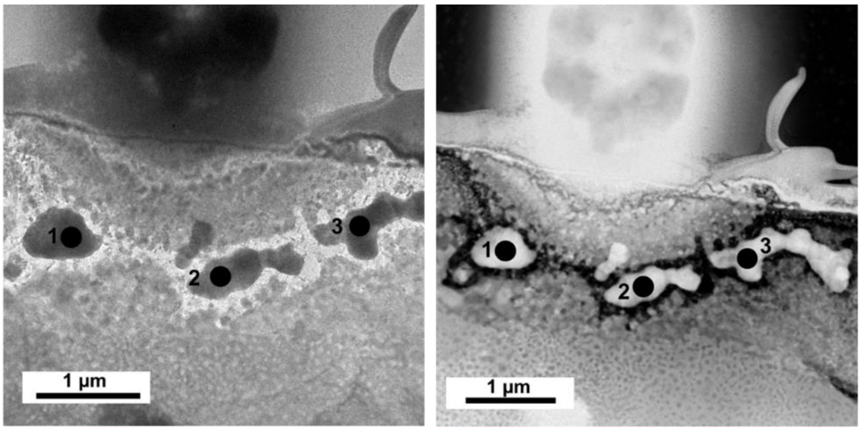

	1	2	3
O	6.7	5.05	5.22
Co	93.30	94.95	94.78
Zr	0	0	0
La	0	0	0
Total	100.0	100.0	100.0

Figure S7. EDS point analyses of the precipitations. In addition to the TEM image on the left hand side, the STEM image in the right hand side shows better contrast between the precipitated Co and Li_2O. EDS show that the precipitation is approximately 95 at% of Co and 5 at% of oxygen after the over-lithiation for the layered-type specimen.

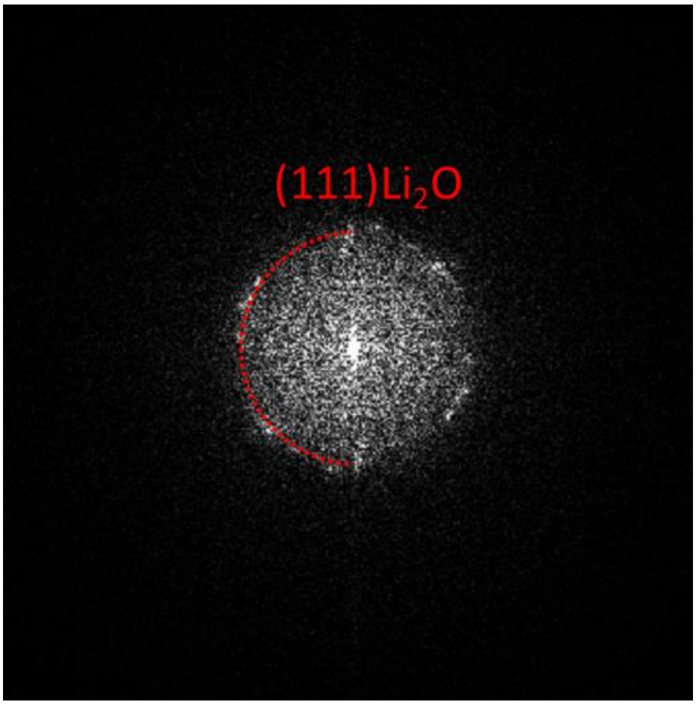

Figure S8. Electron diffraction pattern of polycrystalline Li_2O, which found next to the precipitated Co after the over-lithiation. Polycrystalline electron diffraction ring showing the (111) plane of Li_2O

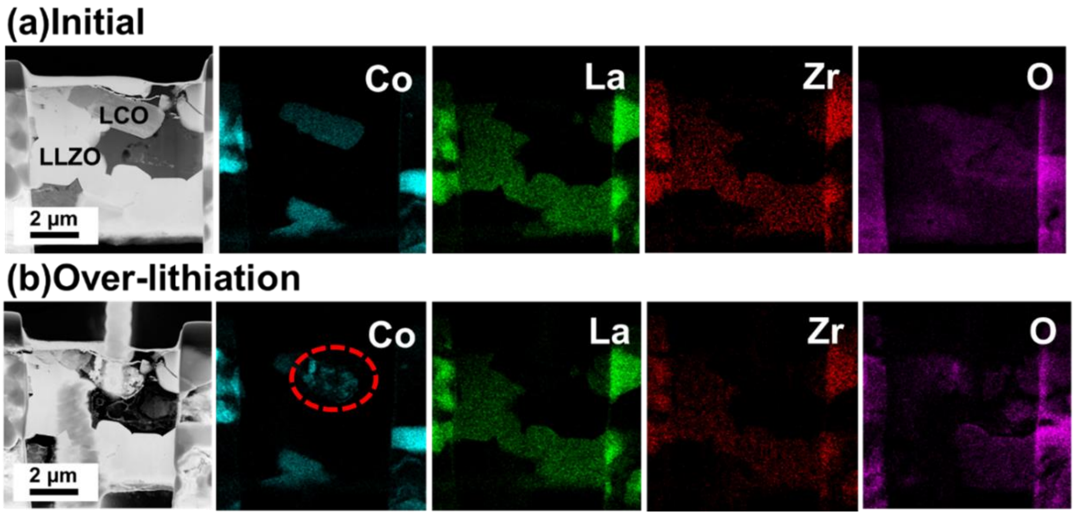

Figure S9. EDS elemental mappings of the pristine and after over-lithiated SSLB sample. The EDS elemental mapping of the (a) pristine, and (b) after over-lithiation. The agglomerates of metallic Co also observed in the sample using CPE.

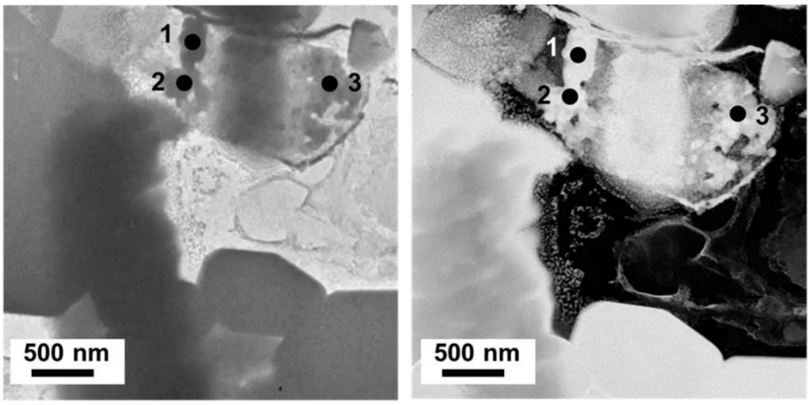

	1	2	3
O	1.23	4.57	7.63
Co	98.77	95.43	92.37
Zr	0	0	0
La	0	0	0
Pt	0	0	0.00
Total	100.00	100.00	100.00

Figure S10. EDS point analyses of the over-lithiated CPE sample. In addition to the TEM image on the left hand side, the STEM image in the right hand side shows better contrast to distinct the precipitated Co. EDS point analyses show that the precipitation is approximately 95at% Co and 5 at% of oxygen after over-lithiation. The results is in agreement with that for Figure S7.

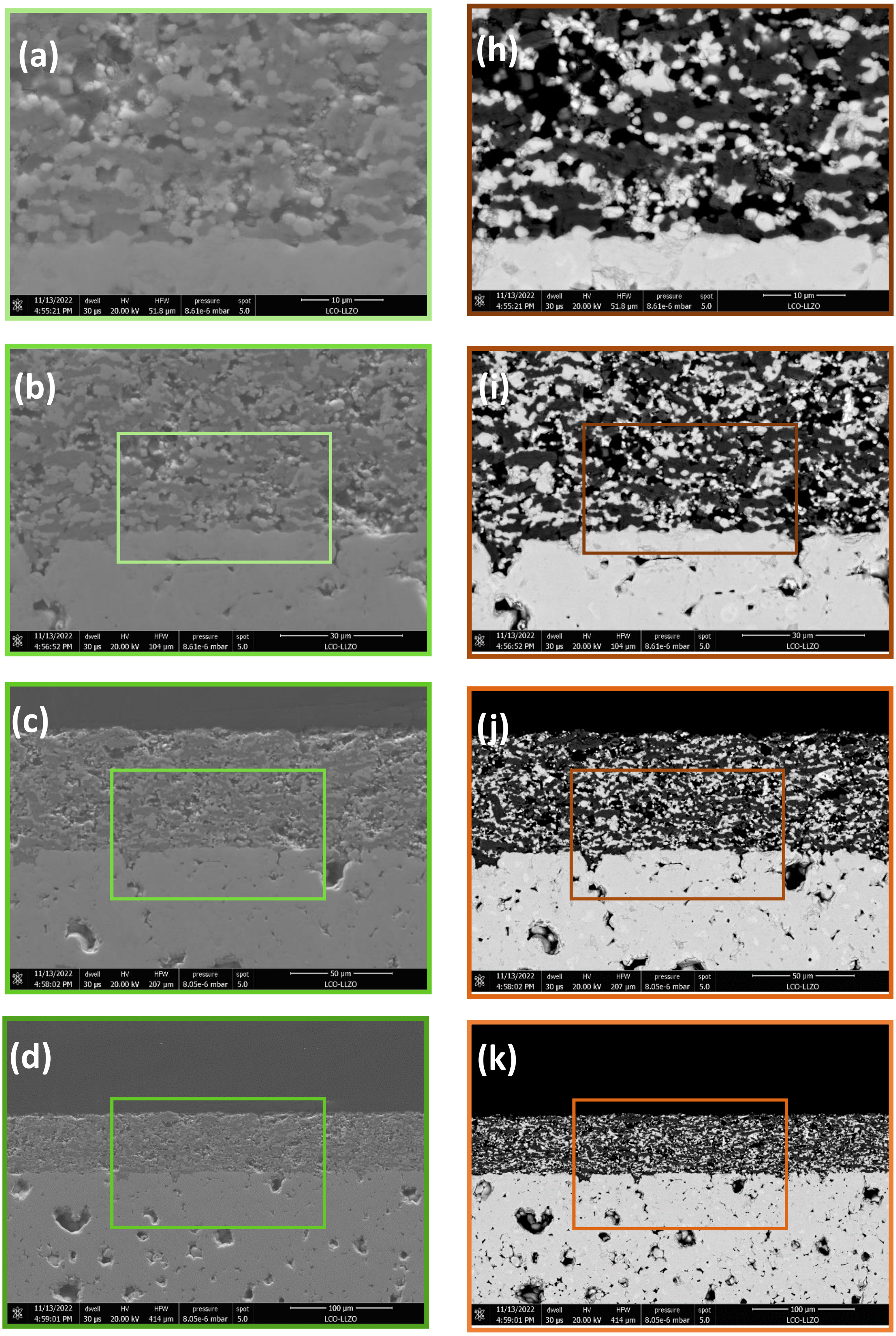
(a)
10 µm
LCO-LLZO
(h)
10 µm
LCO-LLZO
(b)
30 µm
LCO-LLZO
(i)
30 µm
LCO-LLZO
(c)
50 µm
LCO-LLZO
(j)
50 µm
LCO-LLZO
(d)
100 µm
LCO-LLZO
(k)
100 µm
LCO-LLZO

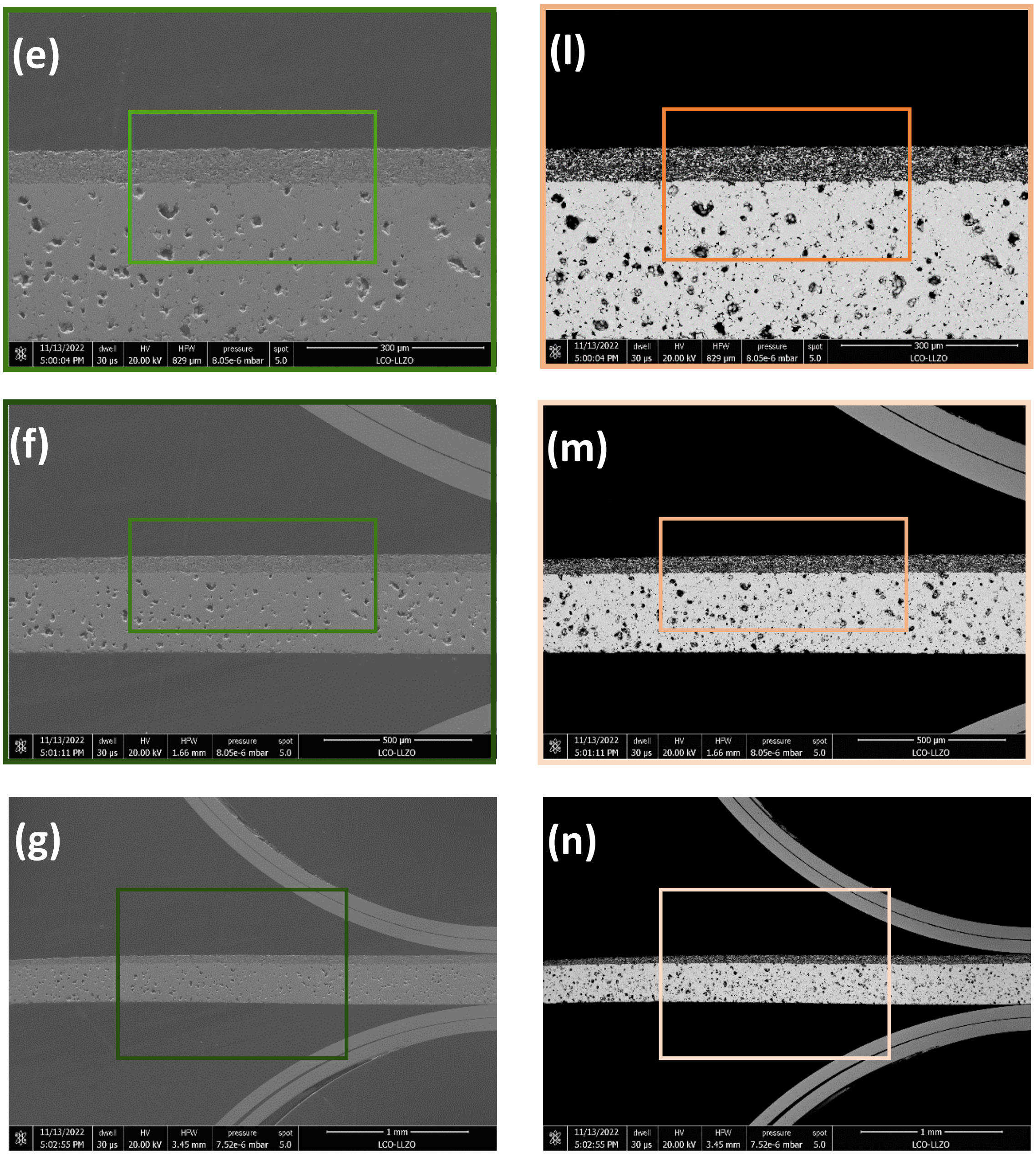

Figure S11. SEM images of SSLB after sintering of CPE onto LLZO. Figure S11 (a)-(g) are SE images and Figure S11 (g)-(n) are BSE images.

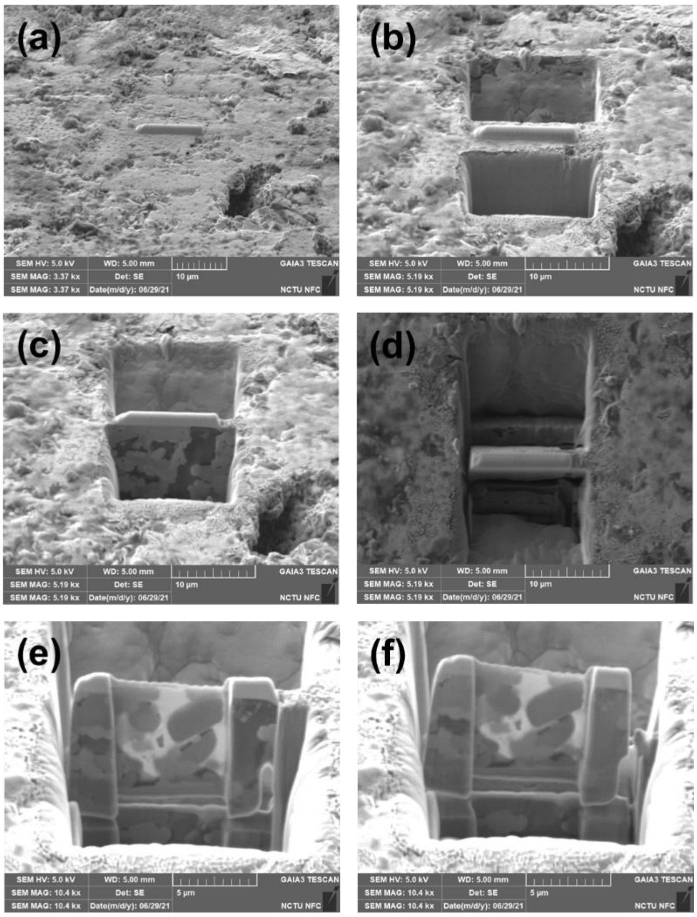

Figure S12. Sample preparation of SSLB by FIB for the *in operando* TEM investigation. (a) Pt deposition, (b) trench, (c) rough polish, (d) U-cut, (e) final polish and (f) cut.

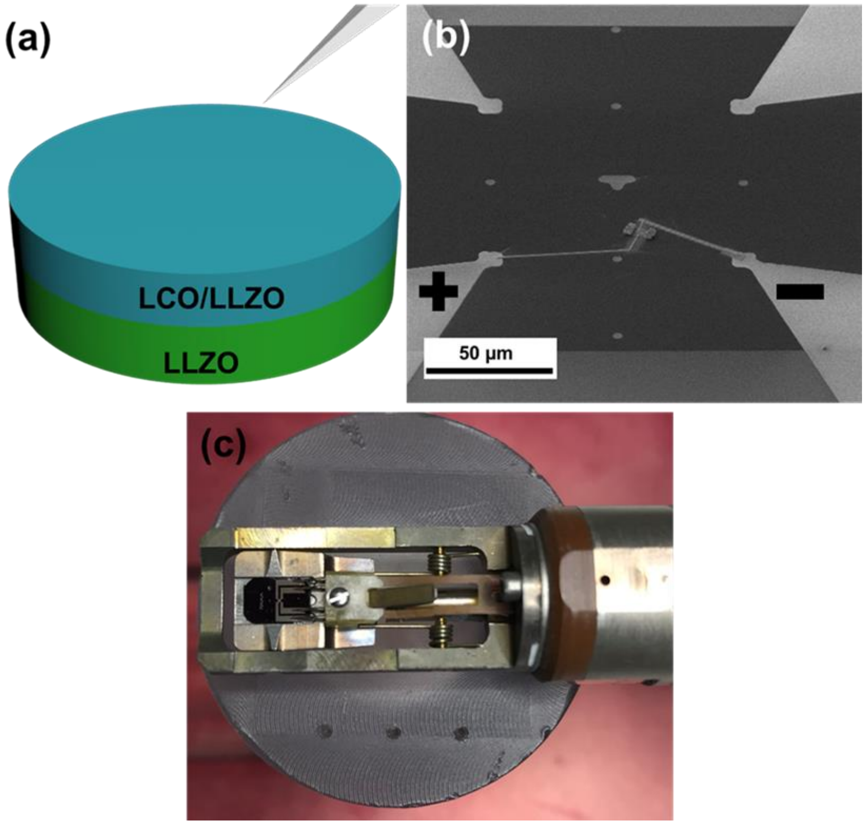

Figure S13. Pt wires deposited by FIB. The fabricated sample is mounted using a glass tip whose schematic is shown in (a). Then, the Pt wires are deposited on the sample by FIB. The SEM image of the entire area is shown in (b). Finally, the sample is transferred to the *in operando* TEM chip as shown in (c).
